# Does Zika Virus Cause Microcephaly - Applying the Bradford Hill Viewpoints

**DOI:** 10.1371/currents.outbreaks.2fced6e886074f6db162a00d4940133b

**Published:** 2017-02-22

**Authors:** Asma Awadh, Abrar Ahmad Chughtai, Amalie Dyda, Mohamud Sheikh, David J. Heslop, Chandini Raina MacIntyre

**Affiliations:** University Of New South WalesUniversity Of New South Wales; University of New South Wales; School of Public Health and Community Medicine, University of New South Wales, Sydney, NSW, Australia; Faculty of MedicineThe University of New South Wales; School of Public Health and Community Medicine, University of New South Wales, Sydney, NSW, Australia

## Abstract

**Introduction:**

Zika virus has been documented since 1952, but been associated with mild, self-limiting disease. Zika virus is classified as an arbovirus from a family of Flaviviridae and primarily spread by Aedes Aegypti mosquitos. However, in a large outbreak in Brazil in 2015, Zika virus has been associated with microcephaly.

**Methods:**

In this review we applied the Bradford-Hill viewpoints  to investigate the association between Zika virus and microcephaly. We examined historical studies, available data and also compared historical rates of microcephaly prior to the Zika virus outbreak. The available evidence was reviewed against the Bradford Hill viewpoints.

**Results:**

All  the nine criteria were met to varying degrees: strength of association, consistency of the association, specificity, temporality, plausibility, coherence, experimental evidence, biological gradient and analogy.

Conclusion: Using the Bradford Hill Viewpoints as an evaluation framework for causation is highly suggestive that the association between Zika virus and microcephaly is causal. Further studies using animal models on the viewpoints which were not as strongly fulfilled would be helpful.

## Background

Until recently, Zika virus has been thought to cause a mild, self-limiting disease. However, in a large outbreak in the Americas in 2015, Zika virus has been implicated as a cause of complications such as microcephaly, Guillaine Barre syndrome (GBS) and myelitis.^1^ Zika virus is classified as an arbovirus from the family of Flaviviridae. It is spread primarily by Aedes Aegypti mosquitoes, and is known to have some neurotropic effects.^2^ The majority of arboviruses cause fever and non-specific symptoms but have been reported to cause severe complications such as neurological, haemorrhagic symptoms, abortions and congenital anomalies in animals and mice.^3^ It was first documented in Rhesus negative monkeys in 1947 and in humans in 1952.^1^ Common symptoms include fever, arthralgia, and rash, however recent evidence has suggested an association with microcephaly.^1^

Microcephaly is defined as babies born with abnormally small skulls and underdeveloped brains relative to gestational age and sex.^4^ In the 2015/16 Zika virus outbreak in Brazil, for the first time there was a reported association with microcephaly.^4^^,^ 5 In Brazil, cases of Zika virus infections began being reported since May 2015. This was followed by reports of an increase in microcephaly cases from November 2015 6.

After experiencing an increase in Zika virus infections, Brazil reported approximately 6158 suspected cases of microcephaly from late 2015, of which 745 cases were confirmed as zika.^7^ In early March, Panama also reported a case of microcephaly related to Zika virus infection.^7^ Previously, in Brazil there were approximately 0.5 cases of microcephaly reported per 10,000 live births, and when rates were calculated using data from the birth defect registry, it was estimated that 1 to 2 cases of microcephaly per 10,000 live births were expected. However, by end of 2015 Brazil had recorded over 3000 babies born with microcephaly, which equates to approximately 20 cases per 10,000 live births.^8^ This rise in incidence in microcephaly prompted the Ministry of Health in Brazil to enhance surveillance, as well as requiring practitioners to report all cases of microcephaly to health departments’ microcephaly registry for further investigation. In addition, a new case definition for microcephaly was adopted to reduce false positive results.^8^

Since this time the association between Zika virus and microcephaly has been debated by leading health agencies, but only a few studies have applied viewpoints for causation in a systematic way.^9^^,^ 10 Frank et al used The Bradford Hill viewpoints to investigate the association between the increased incidence of microcephaly and zika virus infection in pregnancy.^9^ Due to insufficient epidemiological evidence at the time, they suggested causation was not established.^9^ In another study Shephard’s viewpoints of teratogenicity was used to investigate the association of zika virus infection in pregnancy and microcephaly.^10^ Using six of Shepherd’s viewpoints, the authors demonstrated evidence of causation.^10^ This study included more recent evidence compared to the previous one.

The Bradford-Hill Viewpoints are used in public health to determine causation of diseases.^11^ Systematic application of the viewpoints can assist with analysing a possible causal relationship. Additional studies to assess the risk of microcephaly in affected pregnant mothers are ongoing.^12^ In this study we applied the Bradford-Hill viewpoints to investigate the association between Zika virus and Microcephaly.

## Method

Using the Bradford Hills nine viewpoints we analysed data from a 2013 outbreak in French Polynesia and the 2015 Brazilian outbreak to establish a causal link between Zika virus and microcephaly. We searched major databases including PubMed, Medline and Embase from 2000 onwards using search words, such as Zika virus, Microcephaly and Flavivirus. We also searched news reports and grey literature for information on the outbreaks. We examined historical studies and previous data and also we compared historical rates of microcephaly prior to the Zika virus outbreak.

Available data and studies were used to determine whether each of the nine Bradford Hill viewpoints were fulfilled. For strength of association, we sought studies which calculated measures of association for Zika virus and microcephaly. Where the calculation was not done, but suitable data available, we calculated a measure of association. We utilised data from a prospective cohort study conducted in Brazil “Zika virus infection in pregnant women in Rio de Janeiro”,^13^ to calculate an odds ratio (OR) and 95% confidence intervals using a 2x2 table, for microcephaly in ultrasound for women who were Zika virus positive. Due to zero values in the 2x2 table (which makes an OR incalculable) we added 1 to each value in the 2 by 2 table. All analysis was conducted using SPSS 13.0.

The assessment of each viewpoint was qualitative/descriptive, as there was an element of subjectivity in applying quantitative scoring. Evidence collected for each viewpoint is presented in the results with a final judgement as to whether the viewpoint was fulfilled or not.

## Results

**The evidence for each of the Bradford-Hill viewpoints**:

**Viewpoint #1: Strength of association**

Ecological evidence supports the association between Zika virus and microcephaly with several reports from WHO and CDC, illustrating an approximate 20-fold increase in numbers of microcephaly cases in countries which are currently affected with Zika virus outbreak such as Brazil, Colombia, Panama and Martinique.^7^^,^ 8 However, this does not provide quantitative measures of association.

In 2013-2014, Zika virus caused a large outbreak in French Polynesia, where it was estimated to have infected 66% (95% CI 62–70) of the general population.^5^ A well conducted, rigorous retrospective study of this outbreak estimated the probability of Zika virus infection during each week of the epidemic and identified all cases of microcephaly from September 2013 to July 2015.^5^ They developed a model using serological and surveillance data to estimate the proportion of the population infected with Zika virus and create epidemic curves to assess periods for risk during pregnancy and to estimate the associated risk for microcephaly to occur.^5^

Standardized inclusion criteria were used, where all babies born with head circumference of at least 2 standard deviations or below the mean for age, sex and ethnicity of the normal head circumference were considered to have microcephaly. Pregnant women with a clinical picture of Zika infection or with positive PCR for Zika virus were included.^5^ The researchers used medical records of babies born with microcephaly, serological and surveillance data and identified eight cases of microcephaly during the study period, seven of which occurred during the first four months of pregnancy in 2014.^5^ The study found that the estimated risk of microcephaly developing in the fetus was approximately 1% for women infected in the 1st trimester of pregnancy with a risk ratio of 53.4 (95% CI 6.5-1061.2).^5^ The overall risk ratio throughout pregnancy was found to be 20.8 (95% CI 2.1-424.1).^5^

It was also found that the estimated rate of microcephaly in this population during the Zika virus outbreak was substantially higher than the baseline of approximately 2 per 10 000 neonates (0.02%).^5^ The authors note that the 1% risk for microcephaly post-Zika virus infection is low compared with other viruses that cause congenital abnormalities after 1st-trimester infections, such as rubella.^5^ However, the extremely high attack rate of Zika virus infections in a susceptible population can produce a high population burden of congenital effects.^5^

This study found a very strong association between Zika virus infection and microcephaly. With a large sample size from serological and surveillance data obtained from the outbreak, the authors illustrate using mathematical modelling the probability of microcephaly occurrence. The overall risk ratio of 20.8 further strengthens the association between Zika virus and the occurrence of microcephaly in infected pregnant mothers.

In addition, a prospective cohort study was conducted in Brazil with 88 pregnant women with a history of clinical symptoms of Zika virus, out of which 72 women tested positive for the virus.^13^ Among them, 42 women had an ultrasound and 12 of the ultrasounds illustrated fetal abnormalities in comparison to 16 women with negative Zika virus test whose ultrasound were normal.^13^ The above study adds evidence to the association. The inclusion criteria minimise bias in the sample used by only following pregnant women with RT-PCR positive test for Zika virus. One of the limitations is the small sample size used of 88 pregnant women and also the control group of Zika negative mothers was smaller consisting of only 16 pregnant women. Using the data presented in this study, we calculated an odds ratio of 7.13 (95% CI 0.86 to 59.28). This further strengthens the association of outcome of microcephaly in women infected with Zika virus in pregnancy.

The viewpoint is met.

**Viewpoint #2: Consistency of the association**

To date there are two epidemiological studies investigating the association between Zika virus and microcephaly. Of those available studies, there is consistency between studies finding an association. As mentioned above a study conducted using data from the outbreak in French Polynesia in 2013-2014 and a study conducted in Brazil among pregnant women in Rio de Janeiro in 2015 both found a positive association. A case control study in Brazil compared 32 babies born with microcephaly and 62 babies born without microcephaly, for every case born two control cases were matched from the same hospital with area of residence and date of delivery.^14^ This study illustrated a strong association between Zika virus infection in pregnancy and resultant of microcephaly in babies.^14^ Colombia reported a total of 602 cases of microcephaly between EW 1 and EW41 of 2016 which is an increase compared to the expected annual mean of 140 cases per year.^15^ To date there are no available studies that show a negative association between Zika virus and microcephaly. The association has also been demonstrated consistently in different settings, such as Brazil, Colombia and French Polynesia. 5 ^,^ 14 ^,^ 15

The viewpoint is met.

**Viewpoint #3: Specificity**

The criterion for specificity generally refers to one exposure causing one disease. However, when considered more broadly, if there is an association in a specific population group with the same environmental exposure this strengthens the argument for causation.^16^ In this instance the outcome is affecting a specific population (pregnant women) after a specific exposure, which increases the level of evidence supporting the link between Zika virus and microcephaly. 16

This viewpoint should be assessed in combination with the strength of association. Microcephaly is a rare outcome and is estimated to occur in approximately 2 to 12 babies per 10,000 births in the United States of America (USA). The aetiology of microcephaly is usually exposure of the fetus in utero to an external agent or a gene mutation that is capable of causing cell death.^17^^,^ 18 Such causes include the TORCH syndrome (e.g. toxoplasmosis, rubella, cytomegalovirus and herpes), severe malnutrition, chemical or toxic exposures including drugs and alcohol.^17^^,^ 18 In this instance the clinical picture of Zika infection of pregnant women in an area with ongoing Zika outbreak and the outcome of babies with microcephaly suggests specificity of the exposure to Zika virus and the outcome of microcephaly in these areas.^13^ A retrospective study conducted in French Polynesia following a Zkia virus outbreak in 2013-2014 showed 19 cases of microcephaly and four samples of stored amniotic fluid tested positive for the virus.^19^ There are also several other studies which report link between the exposure of Zika virus in pregnant women and microcephaly.^20^^,^ 21 This provides further evidence that exposure to Zika virus can lead to the outcome of microcephaly. This is further illustrated by the isolation of Zika virus in the tissues of foetuses born to Zika positive women and the effect of Zika virus on the progenitor stem cells and impairment of the neurosphere.^18^^,^ 22 ^,^ 23 In this instance the outcome is affecting a specific population (pregnant women) after a specific exposure, which increases the level of evidence supporting the link between Zika virus and microcephaly.^16^

The viewpoint is met.

**Viewpoint #4: Temporality**

In Brazil Zika virus infection was recognised in late 2014 as a febrile illness and the first cases were confirmed by RT-PCR in April 2015, with microcephaly following in November 2015, so that exposure to Zika virus preceded the occurrence of microcephaly.^4^ Martines et al, demonstrated a link between zika virus infection in pregnancy and outcome of microcephaly through detection of viral RNA from brain and placental tissues of fetus of mothers infected with zika virus during pregnancy.^24^ Between 2000 and 2014 Brazil reported an average of 157.3 cases of microcephaly annually. This had increased markedly by end of October 2015 with a total of 26 cases of microcephaly reported registered since August 2015.^4^ A retrospective and prospective review of hospital records from January 2015 to January 2016 illustrates an increase in reported cases of febrile like illness in pregnancy which was clinically diagnosed retrospectively and prospectively using RT-PCR as Zika virus infection.^4^

A study in the State of Paraiba, Brazil, further illustrated the temporality where zika virus was detected in amniotic fluid in two pregnant women with fetal microcephaly.^25^ Analysis of amniotic fluid of the first 31 microcephaly cases in Pernambuco, Brazil, showed the presence of IgM specific antibody for zika virus in the 30 cases, indicating the association of microcephaly with zika virus infection in pregnancy.^26^

A study published in the New England Journal of Medicine (NEJM), reported a possible link between Zika virus and microcephaly which illustrated the temporality. The study, illustrated a case of vertical transmission of Zika virus from a woman in Brazil where she presented with clinical features of Zika virus infection while pregnant. 23 She tested positive for Zika virus infection and ultrasounds done at 14 weeks and 20 weeks showed no indication of foetal anomaly.^23^ But ultrasounds done at 29 weeks showed foetal anomaly and at 32wks microcephaly was diagnosed.^23^ She terminated the pregnancy at 32 weeks and examination of the foetal brain tissue tested positive for Zika virus with the same RNA genome as the circulating Zika virus in Brazil.^23^

It was also reported that a 33-year-old Finish woman became infected while on holiday with her husband in Central America.^22^ She presented with clinical symptoms of Zika virus when she was 4 weeks pregnant and was tested for Zika virus which showed a positive RT-PCR result which persisted up to 10 weeks of pregnancy. (22) The ultrasonography of the fetus showed no effect on the brain in weeks 13,16 or 17, however the fetus’ head circumference was noted to have reduced from the 47th percentile at 16wks to 24th percentile at 20wks and a diagnosis of microcephaly was made.^22^ Another explanation on the persistent positive RT-PCR on the mother is believed to be due to continuous replication of the virus in the foetal brain and placenta.^22^

The epidemiological studies mentioned both show an increase in the number of microcephaly cases following an outbreak of Zika virus.^5^^,^ 13 ^,^ 25 This together with the clear temporal relationship of the epidemic followed by microcephaly births, means the viewpoint for temporality is met.

**Viewpoint #5: Biological gradients or dose-response relationship**

Biological gradients can be illustrated by the level of exposure to a given factor and corresponding level of outcomes.^11^ When a dose–response relationship is found, the evidence for causation is reinforced, however the relationship between exposure and outcome may not be linear.^27^ A Brazilian study reported an increased incidence of babies born with microcephaly in areas affected by zika virus epidemic.^4^ However, the risks of Zika virus leading to congenital abnormalities (1%) is lower than for other virus’ such as rubella (38-100%), cytomegalovirus (13%) and parvovirus B19 (10%).^5^ However, the large outbreak and high attack rate in the population may greatly increase the incidence of Zika virus in pregnant women, which may subsequently lead to a population burden of congenital malformations.^5^ It is also true that countries which are not experiencing an epidemic of zika virus have not seen an increase of microcephaly, whilst those with an active epidemic have. This is indirect evidence of a dose- response relationship.

This viewpoint is met.

**Viewpoint #6: Plausibility**

Flaviviruses in general are known to have some neurotropic effects. The majority of arbovirus cause fever and non-specific symptoms but at times have been shown to cause more severe outcomes such as neurological syndrome and haemorrhagic symptoms, as well as abortion and congenital anomalies in animals and mice.^2^^,^ 3 ^,^ 28 The neuroinvasive and neurovirulent ability has been seen in other flavivirus but to date none has demonstrated ability to cause congenital anomalies except Zika virus.^28^

A case report recently published described a pregnant woman who tested positive for Zika virus and had ultrasounds which indicated malformation in the fetus. Testing of the foetal brain tissue showed the presence of Zika virus as well as the presence of Zika RNA in the placenta and other fetal organs and tissues.^22^ Zika virus has also been isolated in the amniotic fluids of two pregnant women showing its ability to cross the placental barrier.^25^ Transabdominal guided ultrasound amniocentesis in two pregnant women at 28 weeks further shows the ability of the virus to cross the placenta barrier and its potential to affect the neural cells.^25^

Studies conducted to understand the neuropathology of Zika virus illustrate its ability to affect neurons cells and to cause cell death resulting in abnormal head size. This was illustrated by the study of the effect of Zika virus on progenitor cells and immature cortical neurons.^18^^,^ 29 These instances further strengthen the association between the virus and microcephaly. Whilst flavivirus have been previously identified as the cause of neuropathology, this will be the first instance in which a flavivirus is implicated for teratogenicity to date.^18^^,^ 30 There is biological plausibility.

The viewpoint is met.

**Viewpoint #7: Coherence**

The current studies reporting an association between Zika virus and microcephaly do not conflict with previously reported evidence. A recent study conducted in Brazil to demonstrate the effect of Zika virus on the growing neurosphere and brain organoid using human neural stem cells, illustrate the ability of Zika virus to induce death of human neural cells and impair formation of neurosphere hence causing microcephaly.^18^ Another study done on human progenitor human stem cells infected with Zika virus illustrated the ability of the virus to cause increased cell death and cell-cycle dysregulation of embryonic neurones, which slows brain growth.^29^ Many other viruses, including flaviviruses,^31^ are known to be neurotropic, and several infections (such as rubella, CMV and varicella) cause microcephaly and other central nervous system (CNS) malformation.^32^^,^ 33 This adds to coherence with current knowledge for zika virus.^9^^,^ 11 ^,^ 17 Current neuropathology studies as indicated above are consistent with the causal relationship between Zika virus and the development of microcephaly.

The viewpoint is met.

**Viewpoint #8: Experimental evidence**

Scientists working to understand Zika virus have used a cyro-EM structure of mature Zika virus and compared this with other Flavivirus.^1^ This was done by isolating the Zika virus strain from a patient during the French Polynesia outbreak, growing it and then purifying it in mammalian cells.^1^ Later the virus was introduced in to the Vero cell of an Africa monkey for propagation, before being harvested to study the cell structure.^1^ This revealed a detailed structure of the virus which may assist in drug and vaccine development.^1^ The structure appears to be similar to dengue and other Flavivirus with one key difference in variation in its surface enveloped (E) protein, which could be the key in understanding how it enters the cells, and pave the way for antiviral, vaccine and diagnostic test development.^1^ It may also explain the ability of Zika virus to attack nerve cells and cause congenital malformation.^1^

Tang et al, conducted a study using human induced pluripotent stem cells and also on human embryonic stem cells and immature cortical neuron cells to investigate the effect of Zika virus on cells.^29^ He introduce Zika virus from infected monkey cells which was then passed through mosquitoes to human pluripotent cells, embryonic stem cells and immature cortical neuron cells, he then quantifies the infection rate and its effects on these cells.^29^ A spread of infection of about 65-90% was demonstrated on the pluripotent cells within 3days.^29^ The study further showed that the virus ability to infect the embryonic cells and also its ability to produce viral particles, which exhibits the ability to infect immature neurons and replicate itself.^29^ The virus was seen to be able to cause increase cell death and dysregulation in cell cycle which can explain it ability to interfere with neural development in the foetus.^29^

In an experimental study the Brazilian Zika strain was seen to cause microcephaly with other neurodevelopment anomalies in mice models.^34^ The researchers used Zika strain isolated from a case in northeast Brazil then infect pregnant mice and evaluate the newborns pups immediately after they were delivered.^34^ The newborns pups were noted to have growth retardation on physical appearance.^34^ At a cellular level the pups were noted to have cortical malformation with reduced cell numbers and cortical layer thickness was seen to have thinned.^34^ This suggests cell death and cellular dysregulation similar to that seen in the brain tissue of babies born with microcephaly due to zika infection.^34^ This experiment further strengthens the evidence of Brazilian zika strain to have the ability to cause microcephaly and neurodevelopment anomalies.^34^

These experiments further support the association between Zika virus and microcephaly. The above studies illustrated cell structure, infectivity rate and its effects on the human pluripotent stem cells and embryonic cells for better understanding of its ability to induce malformation in neural development. However, more needs to be done in terms of animal models especially in pregnant animals to further elaborate the effects of Zika virus in neural development anomalies.

This viewpoint is met.

**Viewpoint #9: Analogy**

The criteria for analogy was described originally by Hill as analogous examples of exposures that may have led to the same outcome. The examples of thalidomide and rubella were used by Hill as exposures that could each lead to congenital malformations.^27^ Infections that cause the TORCH syndrome^32^^,^ 33, for example, are known to cause abnormalities in the central nervous system, which is analogous to zika virus causing microcephaly.^32^^,^ 35 The cerebral alterations caused by rubella and toxoplasmosis are known^32^^,^ 33 and have some analogy to congenital malformations such as microcephaly described with Zika virus.^36^

In a study done on 151 patients from 2014-2015 in a hospital in Brazil who presented with infection from arbovirus, 6 patients developed autoimmune disorders of which 4 patients had GBS and two patients had Acute demyelinating encephalomyelitis (ADEM).^37^^,^ 38 Brain scans showed damage to white matter in all 6 patients, who also tested positive for Zika virus infection. Five patients reported motor dysfunction, one reported vision problems and one reported a decrease in cognitive functions.^37^^,^ 38 This is further illustrated in Pan American Health Organisation epidemiological report on December 2015 which confirm increase in congenital anomalies like microcephaly in areas which zika virus circulation.^39^

A study was done in Guadeloupe on a 15 year old girl who presented with signs of myelitis and tested positive for Zika infection.^2^ Cerebrospinal fluid of the patient had Zika RNA indicating the neurotropic ability of Zika virus. Other Flavivirus such as Japanese encephalitis virus, Dengue virus and West Nile virus also have neurotropic effects and have been implicated on several instances to cause congenital malformation in babies and central nervous system injury in adults hence can cause alteration of the neural system in pregnant women affecting the unborn babies.^2^^,^ 28 ^,^ 30

The viewpoint is met.

## Discussion

We found that all the Bradford Hill viewpoints were met for the association of microcephaly with zika virus infection, thus adding to the evidence for a causal relationship. The framework of the Bradford Hill Viewpoints is useful for evaluating causation, and can be drawn upon as a systematic method for evaluation of new associations between exposure and disease. Until recently, Zika virus has been generally associated only with a mild febrile illness.^1^^,^ 2 However, this changed during a largescale outbreak in 2015 in South and Central America.^1^ In this outbreak Zika virus infection in pregnant women was associated with an increase in numbers of babies being born with microcephaly and other central nervous system malformations.^1^^,^ 2 ^,^ 8

To date, a small number of studies have been conducted to investigate the possible causal relationship between the outcomes of microcephaly with exposure to Zika virus infection in pregnancy.^5^^,^ 12 ^,^ 13 These studies have provided sufficient evidence regarding the strength of the association between the two, with one study reporting a high relative risk of 20.8.^5^ The consistency criterion is also met with two epidemiological studies conducted in French Polynesia and in Brazil in the 2015-2016 outbreak, both showing the same association.^5^^,^ 13 A temporal relationship is clearly seen in patterns of women being infected with Zika virus in pregnancy and subsequently having babies with microcephaly or/with other neurodevelopment anomalies.^22^^,^ 23 ^,^ 25 ^,^ 26 The specificity of the association is also met. Dose-response relationship (biological gradient) between Zika virus and the outcomes of microcephaly have clearly been met since studies have assessed or quantified the level of viremia and risk of microcephaly. Experimental evidence is present, and flavivirus are known to have neurotropic effects; studies and experiments done on animal models have further illustrated their abilities of neurovirulent and neuroinvasive effects but to date none has been associated with teratogenicity except Zika virus.^3^^,^ 30 ^,^ 40 The current epidemiological and experimental studies on mice models and at cellular level support the link between Zika virus infections in pregnancy and outcome of microcephaly by demonstrating the ability of Zika virus to induce cell death and cell-cycle dysregulation leading to malformation.^18^^,^ 41 ^,^ 42 These studies do not conflict with previously seen evidence in 2013-2014 French Polynesia outbreak where a number of babies were reported to have microcephaly with other congenital anomalies.^5^

Previously two studies have applied the Bradford Hill viewpoints to systematically analyse the evidence for a causal association between Zika infection in pregnancy and microcephaly.^9^^,^ 10 Frank et al, applied the Bradford Hill viewpoints early in the epidemic, but at the time due to lack of epidemiological and molecular studies on Zika virus, they suggested a causal link was not proven.^9^ We have included more recent evidence than was available to Frank et al in their study, which strengthens the evidence of causation.

An alternative approach to the Bradford Hill viewpoints is Shepherd’s criteria of teratogenicity, which have also been used to analyse the link of Zika infection in pregnancy and microcephaly.^10^ One study demonstrated some of the reported evidence to meet the criteria.^10^ The fourth of Shepherd’s criteria requires rare exposure and a resultant rare outcome such as microcephaly. In countries such as Brazil the exposure is widespread, and the outcome rare. The authors of this study defined a rare exposure as travellers to epidemic areas such as Brazil, who acquired infection while pregnant.^10^ In reality, even for a traveller from a non-affected country, the exposure is widespread while travelling in affected areas because of the high attack rates. As such, this criterion has been interpreted very loosely in that study.

Over time since the initial identification of the Brazilian epidemic, more studies and evidence has become available. We systematically applied the accumulated evidence from the available studies to analyse a possible causal relationship between Zika virus infection and microcephaly using Bradford Hill’s viewpoints. The evidence is overwhelmingly in favour of causation. The establishment of causation and increase in certainty aids public health policy and disease control efforts, including travel advisories for pregnant women. In complex outbreaks where causation is unknown, the use of the Bradford Hill viewpoints can assist with systematic decision making and public health policy.

## Competing Interest Statement

The authors have declared that no competing interests exist.

## Data Availability Statement

All data in this review are publicly available in previously published papers, as noted in the Methods section.

## Corresponding Author

Professor C. Raina MacIntyre. Email: r.macintyre@unsw.edu.au

## References

[1] Sirohi D, Chen Z, Sun L, Klose T, Pierson TC, Rossmann MG, et al. The 3.8 Å resolution cryo-EM structure of Zika virus. Science. 2016. 10.1126/science.aaf5316 PMC4845755 27033547

[2] Mécharles S, Herrmann C, Poullain P, Tran T-H, Deschamps N, Mathon G, et al. Acute myelitis due to Zika virus infection. The Lancet. 2016. 10.1016/S0140-6736(16)00644-9 26946926

[3] Hubálek Z, Rudolf I, Nowotny N. Arboviruses pathogenic for domestic and wild animals. Adv Virus Res. 2014;89:201-75. 10.1016/B978-0-12-800172-1.00005-7 24751197

[4] de Oliveira W, Cortez-Escalante J, Holanda De Oliveira W, Ikeda do Carmo G, Henriques C, Coelho G, et al. Increase in Reported Prevalence of Microcephaly in Infants Born to Women Living in Areas with Confirmed Zika Virus Transmission During the First Trimester of Pregnancy — Brazil, 2015. Morbidity and Mortality Weekly Report. 2016;65:242-7. 10.15585/mmwr.mm6509e2 26963593

[5] Cauchemez S, Besnard M, Bompard P, Dub T, Guillemette-Artur P, Eyrolle-Guignot D, et al. Association between Zika virus and microcephaly in French Polynesia, 2013-2015: a retrospective study. The Lancet.387(10033):2125-31 10.1016/S0140-6736(16)00651-6 PMC4909533 26993883

[6] Faria N, da Silva Azevedo R, Kraemer M, Souza R, Cunha M, Hill S, et al. Zika virus in the Americas: Early epidemiological and genetic findings. Science. 2016;352(6283):345-9. 10.1126/science.aaf5036 PMC4918795 27013429

[7] World Health Organisation. Zika Virus; Microcephaly and Guillain Barre Syndrome- Situational Report. 10 March 2016.

[8] Schuler-Faccini L, Ribeiro E, Feitosa I, Horovitz D, Cavalcanti D, Pessoa A, et al. Possible Association Between Zika Virus Infection and Microcephaly — Brazil, 2015. Morbidity and Mortality Weekly Report. January 2016;65:59-62. 10.15585/mmwr.mm6503e2 26820244

[9] Frank C, Faber M, Stark K. Causal or not: applying the Bradford Hill aspects of evidence to the association between Zika virus and microcephaly. EMBO molecular medicine. 2016;8(4):305-7. 10.15252/emmm.201506058 PMC4818755 26976611

[10] Rasmussen S, Jamieson D, Honein M, Petersen L. Zika virus and birth defects—reviewing the evidence for causality. New England Journal of Medicine. 2016;374(20):1981-7. 10.1056/NEJMsr1604338 27074377

[11] Höfler M. The Bradford Hill considerations on causality: a counterfactual perspective. Emerging themes in epidemiology. 2005;2(11). 10.1186/1742-7622-2-11 PMC1291382 16269083

[12] Centers for Disease Control and Prevention. CDC Concludes Zika Causes Microcephaly and Other Birth Defects. 13/04/2016.

[13] Brasil P, Pereira J, Jose P, Raja Gabaglia C, Damasceno L, Wakimoto M, Ribeiro Nogueira RM, et al. Zika virus infection in pregnant women in Rio de Janeiro—preliminary report. New England Journal of Medicine. 2016. 10.1056/NEJMoa1602412 PMC5323261 26943629

[14] de Araújo T, Rodrigues L, de Alencar Ximenes R, de Barros Miranda-Filho D, Montarroyos U, de Melo A, et al. Association between Zika virus infection and microcephaly in Brazil, January to May, 2016: preliminary report of a case-control study. The Lancet Infectious Diseases. 2016. 10.1016/S1473-3099(16)30318-8 PMC7617035 27641777

[15] Pan American Health Organization. Zika-Epidemiological Report, Colombia 3 November 2016 [Available from: http://www.paho.org/hq/index.php?option=com_docman&task=doc_view&gid=35139&Itemid=270&lang=en.

[16] Lucas R, McMichael A. Association or causation: evaluating links between "environment and disease". Bulletin of the World Health Organization. 2005;83(10):792-5. PMC2626424 16283057

[17] Centers for Disease Control and Prevention. Facts about Microcephaly 2016 [Available from: http://www.cdc.gov/ncbddd/birthdefects/microcephaly.html

[18] Garcez P, Loiola E, Madeiro da Costa R, Higa L, Trindade P, Delvecchio R, et al. Zika virus impairs growth in human neurospheres and brain organoids. Science. 2016. 10.1126/science.aaf6116 27064148

[19] Besnard M, Eyrolle-Guignot D, Guillemette-Artur P, Lastere S, Bost-Bezeaud F, Marcelis L, et al. Congenital cerebral malformations and dysfunction in fetuses and newborns following the 2013 to 2014 Zika virus epidemic in French Polynesia. Euro surveillance : bulletin Europeen sur les maladies transmissibles = European communicable disease bulletin. 2016;21(13). 10.2807/1560-7917.ES.2016.21.13.30181 27063794

[20] Jouannic JM, Friszer S, Leparc-Goffart I, Garel C, Eyrolle-Guignot D. Zika virus infection in French Polynesia. Lancet (London, England). 2016;387(10023):1051-2. 10.1016/S0140-6736(16)00625-5 26944027

[21] Pan American Health Organization. Regional Zika Epidemiological Update (Americas) 2016 [Available from: http://www.paho.org/hq/index.php?option=com_content&view=article&id=11599&Itemid=41691&lang=en.

[22] Driggers R, Ho C, Korhonen E, Kuivanen S, Jääskeläinen A, Smura T, et al. Zika Virus Infection with Prolonged Maternal Viremia and Fetal Brain Abnormalities. New England Journal of Medicine.374(22):2142-51. 10.1056/NEJMoa1601824 27028667

[23] Mlakar J, Korva M, Tul N, Popović M, Poljšak-Prijatelj M, Mraz J, et al. Zika virus associated with microcephaly. New England Journal of Medicine. 2016;374(10):951-8. 10.1056/NEJMoa1600651 26862926

[24] Martines R. Notes from the field: evidence of Zika virus infection in brain and placental tissues from two congenitally infected newborns and two fetal losses—Brazil, 2015. Morbidity and Mortality Weekly Report. 2016;65(06):159-60. 10.15585/mmwr.mm6506e1 26890059

[25] Oliveira Melo A, Malinger G, Ximenes R, Szejnfeld P, Alves Sampaio S, Bispo de Filippis A. Zika virus intrauterine infection causes fetal brain abnormality and microcephaly: tip of the iceberg? Ultrasound in Obstetrics & Gynecology. 2016;47(1):6-7. 10.1002/uog.15831 26731034

[26] Cordeiro M, Pena L, Brito C, Gil L, Marques E. Positive IgM for Zika virus in the cerebrospinal fluid of 30 neonates with microcephaly in Brazil. The Lancet. 2016;387(10030):1811-2. 10.1016/S0140-6736(16)30253-7 27103126

[27] Hill A. The environment and disease: association or causation? Proceedings of the Royal society of Medicine. 1965;58(5):295. 10.1177/003591576505800503 PMC1898525 14283879

[28] Sips G, Wilschut J, Smit J. Neuroinvasive flavivirus infections. Reviews in medical virology. 2012;22(2):69-87. 10.1002/rmv.712 22086854

[29] Tang H, Hammack C, Ogden S, Wen Z, Qian X, Li Y, et al. Zika virus infects human cortical neural progenitors and attenuates their growth. Cell stem cell. 2016;18(5):587-90. 10.1016/j.stem.2016.02.016 PMC5299540 26952870

[30] Nagata N, Iwata-Yoshikawa N, Hayasaka D, Sato Y, Kojima A, Kariwa H, et al. The Pathogenesis of 3 Neurotropic Flaviviruses in a Mouse Model Depends on the Route of Neuroinvasion After Viremia. Journal of Neuropathology & Experimental Neurology. 2015;74(3):250-60. 10.1097/NEN.0000000000000166 25668565

[31] Gould EA, Solomon T. Pathogenic flaviviruses. Lancet (London, England). 2008;371(9611):500-9. 10.1016/S0140-6736(08)60238-X 18262042

[32] Edlich R, Winters K, Long III W. Rubella and congenital rubella (German measles). Journal of long-term effects of medical implants. 2005;15(3):319-28. 10.1615/jlongtermeffmedimplants.v15.i3.80 16022642

[33] Goldenberg R, Culhane J, Johnson D. Maternal infection and adverse fetal and neonatal outcomes. Clinics in perinatology. 2005;32(3):523-59. 10.1016/j.clp.2005.04.006 PMC7119141 16085019

[34] Cugola F, Fernandes I, Russo F, Freitas B, Dias J, Guimarães K, et al. The Brazilian Zika virus strain causes birth defects in experimental models. Nature. 2016;534(7606):267-71. 10.1038/nature18296 PMC4902174 27279226

[35] Calvet G, Aguiar R, Melo A, Sampaio S, de Filippis I, Fabri A, et al. Detection and sequencing of Zika virus from amniotic fluid of fetuses with microcephaly in Brazil: a case study. The Lancet Infectious Diseases.16(6):653-60. 10.1016/S1473-3099(16)00095-5 26897108

[36] Hazin A, Poretti A, Di Cavalcanti Souza Cruz D, Tenorio M, van der Linden A, Pena L, et al. Computed Tomographic Findings in Microcephaly Associated with Zika Virus. New England Journal of Medicine. 2016;374(22):2193-5. 10.1056/NEJMc1603617 27050112

[37] Brito C. Zika virus: a new chapter in the history of medicine. Acta medica portuguesa. 2016;28(6):679-80. 10.20344/amp.7341 26849748

[38] American Academy of Neurology. Zika Virus May Now Be Tied to Another Brain Disease 2016 [Available from: https://www.aan.com/PressRoom/home/PressRelease/1451

[39] Pan American Health Organization. Epidemiological Alert . Neurological syndrome, congenital malformations, and Zika virus infection. Implications for public health in the Americas 1 December 2015 [Available from: http://www.paho.org/hq/index.php?option=com_docman&task=doc_view&Itemid=270&gid=32405&lang=en.

[40] Fernandez-Garcia M, Mazzon M, Jacobs M, Amara A. Pathogenesis of flavivirus infections: using and abusing the host cell. Cell host & microbe. 2009;5(4):318-28. 10.1016/j.chom.2009.04.001 19380111

[41] Lazear H, Govero J, Smith A, Platt D, Fernandez E, Miner J, et al. A mouse model of Zika virus pathogenesis. Cell Host & Microbe. 2016;19(5):720-30. 10.1016/j.chom.2016.03.010 PMC4866885 27066744

[42] Li C, Xu D, Ye Q, Hong S, Jiang Y, Liu X, et al. Zika Virus Disrupts Neural Progenitor Development and Leads to Microcephaly in Mice. Cell Stem Cell. 2016;1(120-6). 10.1016/j.stem.2016.04.017 27179424

